# Superior Canal Dehiscence Syndrome: Lessons from the First 20 Years

**DOI:** 10.3389/fneur.2017.00177

**Published:** 2017-04-28

**Authors:** Bryan K. Ward, John P. Carey, Lloyd B. Minor

**Affiliations:** ^1^Department of Otolaryngology-Head and Neck Surgery, Johns Hopkins University School of Medicine, Baltimore, MD, USA; ^2^Department of Otolaryngology-Head and Neck Surgery, Stanford University School of Medicine, Stanford, CA, USA

**Keywords:** superior semicircular canal dehiscence syndrome, vestibular diseases, autophony, vertigo, labyrinth diseases

## Abstract

Superior semicircular canal dehiscence syndrome was first reported by Lloyd Minor and colleagues in 1998. Patients with a dehiscence in the bone overlying the superior semicircular canal experience symptoms of pressure or sound-induced vertigo, bone conduction hyperacusis, and pulsatile tinnitus. The initial series of patients were diagnosed based on common symptoms, a physical examination finding of eye movements in the plane of the superior semicircular canal when ear canal pressure or loud tones were applied to the ear, and high-resolution computed tomography imaging demonstrating a dehiscence in the bone over the superior semicircular canal. Research productivity directed at understanding better methods for diagnosing and treating this condition has substantially increased over the last two decades. We now have a sound understanding of the pathophysiology of third mobile window syndromes, higher resolution imaging protocols, and several sensitive and specific diagnostic tests. Furthermore, we have a treatment (surgical occlusion of the superior semicircular canal) that has demonstrated efficacy. This review will highlight some of the fundamental insights gained in SCDS, propose diagnostic criteria, and discuss future research directions.

## Introduction

In 1998, Minor et al. described a series of patients with symptoms of chronic disequilibrium and sound- or pressure-induced vertigo and nystagmus in the plane of the superior semicircular canal (1). Computed tomography (CT) imaging revealed a bony dehiscence over the superior semicircular canal in these patients, and a few underwent surgery to plug and resurface the superior semicircular canal, after which the primary symptoms improved. As additional patients were recognized, symptoms of bone conduction hyperacusis (i.e., hearing internal noises transmitted loudly to the affected ear) and pulsatile tinnitus became prominent features (2). The name superior canal dehiscence syndrome (SCDS) was used to describe patients with these unique symptoms associated with the presence of a bony dehiscence over the superior semicircular canal.

The syndrome has subsequently been modeled as a third mobile window in the labyrinth (3). Sound pressure entering the oval window *via* the stapes normally exits at the elastic round window. Superior canal dehiscence presents a novel low-impedance pathway for pressure entering at the oval window to dissipate through the labyrinth instead of the cochlea. For air-conducted sound, the result is a loss of energy and corresponding increase in thresholds for hearing. However, for bone-conducted sound, the opposite is true: the low impedance of the dehiscence permits bone-conducted sound to access the perilymph of the inner ear *via* the labyrinth, and the free communication of the perilymph with the cochlea results in hearing bone-conducted sounds better than normal. This “bony hyperacusis” manifests as symptoms of autophony (hearing one’s own voice as loud or distorted); pulsatile tinnitus; and audible eye movements, footsteps, chewing, bowel movements, etc. The laboratory correlates are not only audiometric air-bone gaps, but characteristic negative bone conduction thresholds; yet, stapedial reflexes remain intact, a contradistinction from the characteristics of conductive hearing loss due to fixation of the stapes or other ossicles (4, 5). In addition, pressure gradients between the oval window and dehiscence cause flow of endolymph in the superior canal ampulla, causing vertigo and nystagmus corresponding to either excitation or inhibition of the superior canal. These pressure gradients can be generated in the ampullofugal (excitatory) direction by loud sound, positive pressure applied to the external auditory canal, or Valsalva against pinched nostrils. Conversely, ampullopetal (inhibitory) flow results from increases in intracranial pressure (e.g., Valsalva against closed glottis) or negative pressure applied to the external auditory canal (6). The third mobile window model has support not only from considerable clinical experience (7–9), including improvement or resolution of symptoms and signs with surgery that occludes and seals the bony defect, but also a growing experimental evidence as well (10–12).

**Figure 1:**
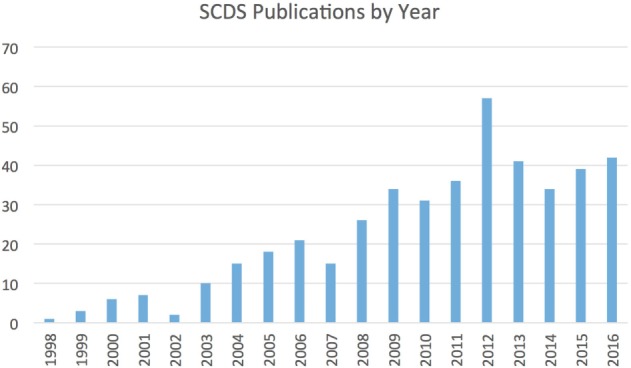
**Number of publications related to SCDS by year since its original description**. Figures derived from PubMed search for “superior canal dehiscence.”

Since the first publication, over 600 cases of SCDS have been reported, and research productivity directed at understanding better methods for diagnosing and treating this condition has substantially increased (Figure 1). This review will highlight some of the fundamental insights gained in SCDS, propose diagnostic criteria, and discuss future research directions.

## Historical Context

**Figure 2:**
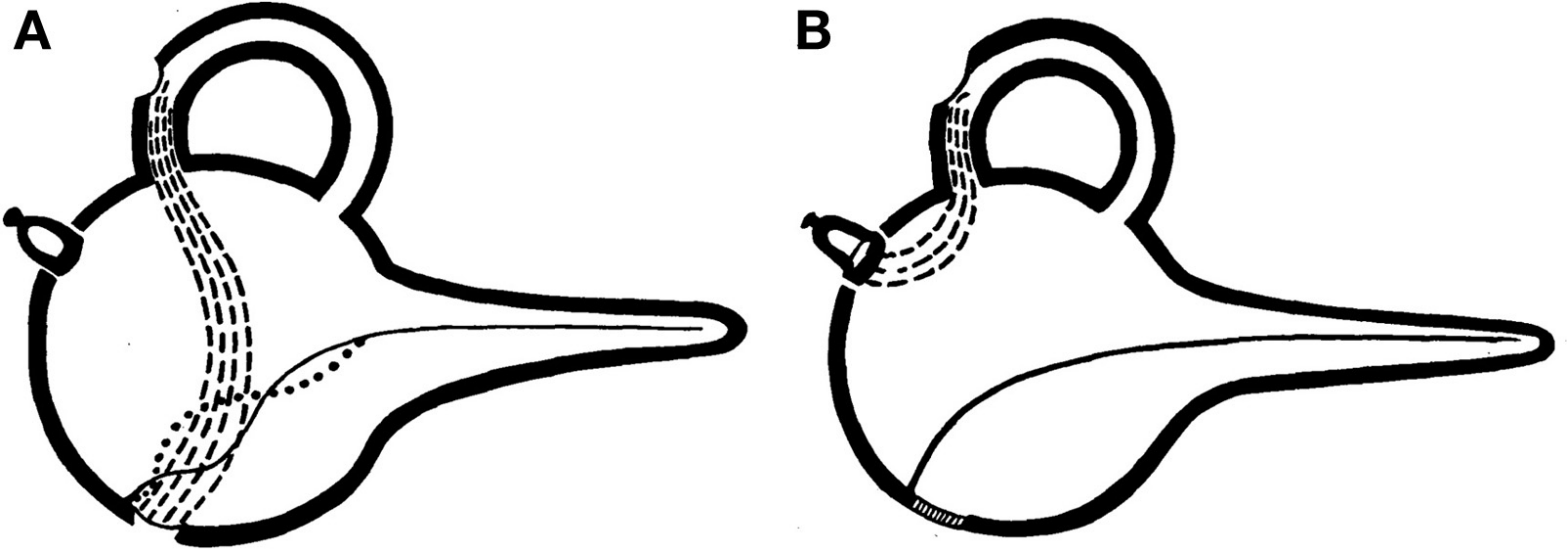
**Original graphical demonstrations by Cawthorne of the third mobile window effect**. **(A)** Demonstrates the effect of semicircular canal fenestration in otosclerosis with a fixed stapes footplate and two mobile windows and **(B)** third mobile window established *via* semicircular canal fenestration in a patient with a mobile stapes footplate.

Symptoms of sound-induced vertigo or dizziness have been recognized as a clinical complaint of patients related to the labyrinth for at least 70 years (13). In the early twentieth century Pietro Tullio observed in pigeons that when a hole was made in a semicircular canal, the labyrinth became responsive to externally applied sound stimuli, inducing eye and head movements in the plane of the fenestrated canal (14, 15). Subsequent work by Huizinga attributed this observation to creating a new low-resistance pathway through the inner ear (16). Cawthorne described Tullio’s phenomenon in patients who had undergone fenestration procedures for otosclerosis in which the stapes was not fixed, creating a “third window” in the inner ear (17), Figure 2. Hennebert also identified patients with congenital syphilis in whom pressure applied to the ear canal produced vestibular symptoms and signs (18). We now commonly apply the term Tullio phenomenon to the symptom of vertigo in response to loud sound and Hennebert sign to the same symptoms and eye movements in response to an externally applied pressure at the ear canal.

David Robinson’s development of the magnetic scleral search coil allowed both improved accuracy of eye movement measurements and the recording of three-dimensional eye movements (19). With increased attention directed to observing eye movements in patients with Tullio phenomenon, several groups identified patients with vertical and torsional eye movements in response to loud sounds or pressure (20–22). A key insight that led to the discovery of SCDS was observing that when patients were exposed to pressure changes or loud sounds they had eye movements in the plane of the ipsilateral superior semicircular canal, linking their symptoms to anatomy. High-resolution CT imaging revealed a dehiscence in the bone over the superior semicircular canal in these patients, creating a “third window” as described by Cawthorne (1).

## Etiology

Evolutionary adaptations have allowed the auditory and vestibular organs to maintain close proximity yet functional independence (23). The presence of a dehiscence can disturb this independence, leading both to alterations in the way sounds are transmitted to the ear and to vertigo in response to sound. The underlying pathophysiology of SCDS is the presence of a third mobile window in the inner ear, in addition to the oval and round windows (1). As a result of a dehiscence in the otic capsule, inner ear biomechanics are altered, such that low-frequency acoustic stimuli of high intensities may create a traveling wave toward the dehiscence, stimulating the vestibular end organs (3, 24). This shunting of acoustic energy creates both a distortion to sound perception, causing hyperacusis and reverberation, and sound- and pressure-evoked vertigo and dizziness (10, 25).

The etiology of SCDS is unknown, but it appears not to be cephalic displacement of the labyrinth during development (26). Currently, there are two primary theories: congenital and acquired. Based on the results of a large temporal bone study at Johns Hopkins (27), we believe SCDS is primarily a congenital phenomenon. From that study, temporal bones that showed thinning or dehiscence over the superior semicircular canal did not have evidence of bony remodeling except in rare cases (i.e., one patient with a meningioma). Others have found similar results in a patient who had confirmed SCDS during life (28). While otic capsule bone differs from the rest of the skeleton in having low bone turnover, the otic capsule is thought to develop for several years after birth. Many groups have observed that the prevalence of a dehiscence on CT is high in infants but decreases during the first decade of life (29–32), providing some support for the congenital theory. SCDS affects both ears in about 25% of patients, also consistent with congenital predisposition (33). Although there are a few cases of familial SCDS (34, 35) and a new report that indicates a high prevalence of canal dehiscence in patients with CDH23 variants (Usher syndrome type 1D) (36), strong genetic correlates have not been identified.

In as many as one quarter of cases, however, another inciting injury such as a traumatic head injury or Valsalva initiates symptoms (37). Many surgeons have noticed that patients with SCDS often have numerous tegmen defects (2, 38–40) as well as a dehiscent geniculate ganglion (41, 42), with some groups arguing that this supports a congenital etiology, and a few to speculate that over many years the slow pulsations of the brain and cerebrospinal fluid that surrounds it may lead to the development of both SCDS and tegmen defects (43). Intracranial hypertension has been hypothesized as contributing to SCDS; however, patients with SCDS tend not to be obese (44), suggesting obesity is not a mediator in the development of SCDS. Supporting the acquired theory is the observation on CT imaging of increasing thinning of the bone over the superior semicircular canal with advanced age (45, 46) and a few cases in which radiographic progression has been observed (47). Unusual cases of abnormalities of the middle and posterior cranial fossae have been identified as causing cases of SCDS (48–51), but these are exceptional.

Aside from dehiscence of the superior semicircular canal, several other sources of labyrinthine dehiscence can lead to symptoms similar to those in SCDS, including that of the posterior semicircular canal (52, 53), lateral semicircular canal (17, 54), vestibular aqueduct (55), facial nerve (56), internal auditory canal (57), and the carotid canal (58, 59). Merchant and Rosowski synthesized many of these reports and broadly proposed that any dehiscence of the inner ear can lead to an inner ear conductive hearing loss from a third mobile window (60). Beyond frank dehiscence, however, we have suggested that focal thinning—perhaps accompanied by pinpoint dehiscences—of otic capsule bone can in some cases transmit pressure and cause symptoms of SCDS (61), and others have suggested that a more global thinning in some conditions such as Paget’s disease (60, 62) can lead to similar phenomena. Nakajima and colleagues have emphasized that any opening, even pinpoint ones, can sufficiently alter the impedance of the otic capsule to cause a functional third mobile window (12, 63).

## Symptoms

The most common symptoms of SCDS include bone conduction hyperacusis, autophony, pulsatile tinnitus, and sound- or pressure-induced vertigo (2, 37). Some of the internal noises that patients report as being particularly disturbing include hearing their eyeballs move, hearing their footfalls loudly, chewing, belching, or borborygmi. Patients also experience aural fullness. Chronic disequilibrium is common, and many patients with SCDS often report a sensation of “brain fog” that may be related to vestibular contributions to attention and other aspects of cognition (64). Patulous Eustachian tube dysfunction can also present with autophony, voice distortion, and pulsatile tinnitus (65). In patulous Eustachian tube dysfunction, hearing one’s nasal breathing and symptom relief when in supine position are commonly thought to be distinguishing features; however, although breath autophony is uncommon in SCDS, half of patients with SCDS may experience symptom relief when supine (66).

Many patients with SCDS also have migraine, but this may represent the high prevalence of migraine in the general population and that SCDS is an effective migraine trigger. Some unusual symptoms have included a patient with tinnitus with head movements in the plane of the affected semicircular canal (67), as well as vertical head movements when hearing a loud sound. The vestibular system influences reflexes that control the neck musculature, as evidenced by the early vestibular physiology studies performed in pigeons and referenced above. It is therefore particularly interesting that a few patients can develop involuntary head movements in response to loud sounds, and that these movements occur in the plane of the superior semicircular canal (6). Vestibular contributions to the muscles controlling head movements may explain the neck muscular strain reported by some patients with SCDS.

Whether SCDS is progressive is unclear. It appears that the hearing loss does not significantly change over time (68). There have been reported cases of worsening conductive hearing loss over time and cases have been reported in which symptoms progressed over many years (35, 47, 69), while at least one case developed rapid mixed hearing loss (70). If SCDS is related to a congenital predisposition, patients may develop worsening symptoms as the dehiscence becomes larger with increasing age. As a result, pediatric patients may present differently than adults (69). Despite the high prevalence of an anatomic dehiscence noted on CT in young children (described above), only a few cases of pediatric SCDS has been reported (71), even fewer of whom underwent surgical repair (72).

## Diagnosis

### Imaging

Computed tomography imaging demonstrating a dehiscence is an important diagnostic feature of SCDS, but it is not sufficient for diagnosis and may mislead the ordering physician. On review of 1,000 temporal bones, the prevalence of a dehiscent superior semicircular canal is 0.5% (27), yet as many as 9% of patients may have a dehiscence on a coronal temporal bone CT with 1-mm slice thickness (73). Higher resolution studies can improve diagnostic accuracy. For the diagnosis of SCDS, temporal bone CT images should be obtained with slice thickness less than 1 mm (ideally 0.625 mm or less) and reformatted in the planes of the superior semicircular canal (Pöschl view) and orthogonal to it (Stenvers view). Due to volume averaging and other factors, however, CT imaging can still overcall a dehiscence (74). Furthermore, many patients with CT evidence of a dehiscence are asymptomatic, perhaps reflecting the protective role of inelastic dura in preventing pressure transmission through some bony dehiscences. In addition to a dehiscence of CT imaging, therefore, patients must also have both symptoms consistent with the syndrome and physiological evidence of a third mobile window.

Magnetic resonance imaging (MRI) has been explored as a possible alternative to CT for diagnosis (75, 76); and some centers routinely perform MRI (in addition to CT) to better evaluate the skull base for vascular malformations, masses, or encephaloceles prior to surgery. It has been our preference to reserve MRI only for cases of persistent symptoms after an initial attempt at surgery. If an MRI is performed, the best sequences for viewing the semicircular canals are heavily T2 weighted and have a variety of names depending on the MRI manufacturer (e.g., FIESTA, CISS). In these sequences, semicircular canal fluid signal is bright; loss of this signal can be useful for assessing adequacy of prior surgical plugging (77).

### Clinical Exam

Fortunately, for diagnostic purposes a variety of abnormal physiologic findings have been observed in SCDS that can provide evidence of a third mobile window. In many of the first patients described by Minor et al., eye movements or nystagmus in the plane of the superior semicircular canal were observed, a critical finding that led to localizing the source of symptoms to the superior semicircular canal (6). In our practice, this test is performed during the clinic visit using an audiometer during which a range of different frequency tones are played to the ear at varying intensity while monitoring the patient’s eye movements with video-oculography or Frenzel lenses. This finding is not observed in all patients (2), and when observed is not always in the plane of the superior semicircular canal. When the Tullio phenomenon elicits eye movements not in the plane of the superior semicircular canal, however, clinicians should consider alternative diagnoses due to the rarity of this finding. As mentioned above, approximately 20% of patients have head movements in the plane of the superior semicircular canal during this evaluation. Paradoxically, patients with larger dehiscence length (typically >5 mm) also can have impaired function of the affected superior semicircular canal due to “autoplugging” in which temporal dura herniates through the dehiscence and compresses the membranous duct (6).

### Pure Tone Audiometry

On pure tone audiometry, one of the more common findings is a large air-bone gap at the lower frequencies (250, 500, and 1,000 Hz). As a result, many early cases were suspected of having otosclerosis; it is important to perform acoustic reflexes, as these are commonly normal in SCDS. There have been a few cases reported in which patients have both otosclerosis and SCDS, but these cases are atypical (78–80). Increased dehiscence length has been shown to correlate with larger air-bone gaps (63, 81), and this is predicted on modeling of a dehiscence as well (63). In many patients, the bone conduction threshold at these frequencies is negative or better than normal. In order to capture this, however, audiometers must be calibrated appropriately, and audiologists need to be aware of the need to test bone conduction thresholds below 0 dB hearing level. A clinical assessment that is oftentimes confirmatory of negative bone conduction thresholds is Weber tuning fork testing, in which a struck 512 Hz tuning fork will be heard more loudly in the ear with greater bone conduction hyperacusis (i.e., negative thresholds). Sometimes, the tuning fork can be heard in the affected ear when placed on the medial malleolus or other distant bony prominences (82).

### Vestibular-Evoked Myogenic Potentials (VEMPs)

Vestibular-evoked myogenic potentials are electromyographic potential reflex tests that in the non-dehiscent ear are thought to reflect function of the saccule (cervical VEMP) or utricle (ocular VEMP) (83). The cervical VEMP involves an inhibitory neural reflex pathway from the saccule to the ipsilateral sternocleidomastoid muscle. The ocular VEMP involves an excitatory pathway from the utricle to the contralateral inferior oblique muscle. In SCDS, these tests are frequently abnormal, as the affected ear is especially sensitive to the auditory or vibratory stimuli used to evoke these myogenic potentials. Patients with SCDS frequently have lower than normal thresholds for cervical VEMP responses to an audible click or tone burst and elevations in the ocular VEMP amplitude responses. Ocular VEMP amplitudes in particular have been found to be highly sensitive and specific for the diagnosis of an intraoperative confirmed dehiscence (84, 85). We therefore believe VEMPs to be an essential component to the diagnostics of SCDS.

### Electrocochleography (ECoG)

Electrocochleography was formerly a popular test for endolymphatic hydrops associated with Meniere’s disease. Arts et al. identified that patients with SCDS consistently have elevations in the summating potential (SP) to action potential (AP) ratio, and that this abnormality corrects after surgical plugging of the affected canal (86, 87). These findings have subsequently been observed by us and others (88, 89). While the results have not yet correlated with postoperative hearing outcomes, changes such as rapid rises in the SP are often observed during surgery and likely reflect changes in inner ear biomechanics during vestibular surgery. The clinical utility of this test for diagnosis and intraoperative use is still under investigation; nevertheless, ECoG appears to reflect the presence of a third mobile window, similar to the other diagnostic testing described above.

### Diagnostic Criteria

**Figure 3:**
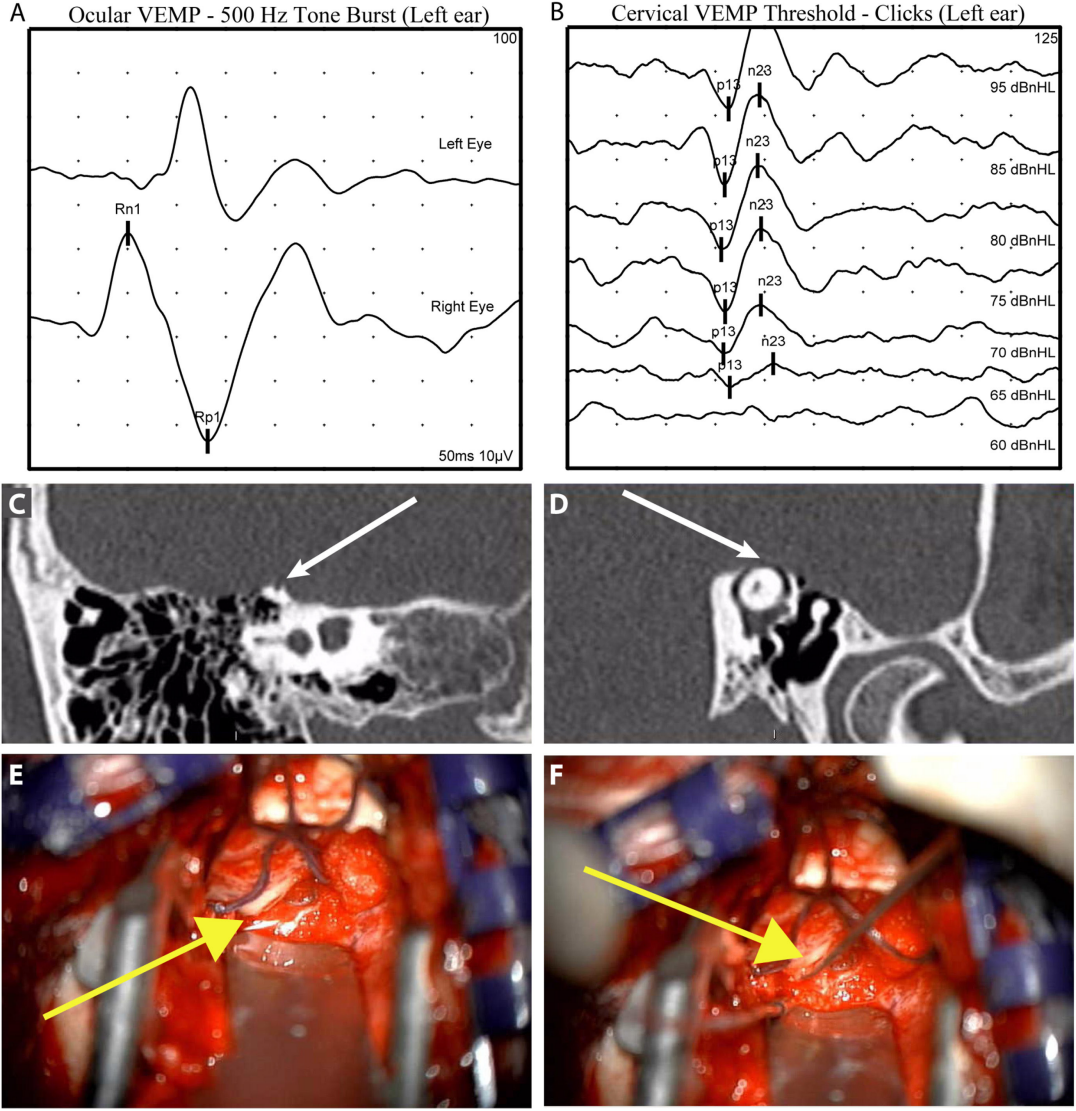
**Illustrative case to demonstrate diagnostic and intraoperative findings: a 40-year-old man presented with 6 years of left aural fullness, pulsatile tinnitus, vocal distortion, and hearing his eyeballs move in his left ear**. Ocular vestibular-evoked myogenic potentials (VEMPs) indicated elevated amplitude responses to 500 Hz tone bursts [**(A)**, 47.3 µV, normal range 0–17 µV] and cervical VEMPs with low thresholds in response to clicks [**(B)**, 65 dB nHL, normal range ≥80 dB nHL], both suggestive of a third mobile window syndrome involving the left ear. High-resolution computed tomography imaging with 0.6-mm slice thickness demonstrated a dehiscence of the left superior semicircular canal when image reconstructions were made orthogonal to the plane of the superior canal [**(C)**, Stenvers view] and in the plane of the superior canal [**(D)**, Pöschl view]. He elected to proceed with surgery *via* middle cranial fossa approach. The dehiscence measured 5 mm × 1 mm [**(E)**, yellow arrow] and was plugged with a combination of autologous materials including fascia, bone dust, and bone chips **(F)**. The middle cranial fossa was resurfaced with hydroxyapatite cement. Autophony improved after surgery, hearing was preserved, and vestibular dysfunction was limited to the superior semicircular canal as determined by clinical head impulse testing in all semicircular canal planes.

The diagnosis of SCDS is based on the combination of CT evidence of a dehiscence, patient symptoms, and evidence of abnormal pressure transmission *via* a third mobile window (see Figure 3, for an example case). We synthesized the following diagnostic criteria for superior semicircular canal dehiscence syndrome based on patients from our institution who had SCDS, underwent surgery, and reported improvement in symptoms (Table 1).

**Table 1:** **Proposed diagnostic criteria for superior canal dehiscence syndrome (SCDS)**.

Patients should meet the following conditions: 1.High-resolution computed tomography images (≤0.625-mm slice thickness) reformatted in the plane of the superior SCC demonstrating a dehiscence 2.At least one of the following symptoms consistent with SCDS A.Bone conduction hyperacusis (in the form of autophony, audible eye movements, audible footsteps, etc.) B.Sound-induced vertigo C.Pressure-induced vertigo (*via* nasal or glottic Valsalva or pressure applied to the external auditory canal) D.Pulsatile tinnitus 3.At least one of the following diagnostic tests indicating a third mobile window A.Negative bone conduction thresholds on pure tone audiometry B.Enhanced VEMP responses (low cervical VEMP thresholds or high ocular VEMP amplitudes) C.Elevated summating potential to action potential ratio on electrocochleography in the absence of a sensorineural hearing loss

*SCC, semicircular canal; VEMP, vestibular-evoked myogenic potential*.

*VEMP thresholds should be compared to laboratory norms*.

## Treatment

### Canal Plugging and Resurfacing

There are no known effective medical treatments for SCDS. While some patients with SCDS are content to have an explanation for their symptoms, some (about half in our experience) pursue surgery. We offer surgery to patients when we can relate their symptoms to SCDS and when the patient can tell themselves that their symptoms are debilitating. As part of the original series on SCDS reported by Minor et al., a few patients underwent surgical resurfacing and/or plugging of the superior semicircular canal by middle cranial fossa approach and experienced resolution of symptoms (1). The goal of surgery is elimination of the third mobile window pathophysiology. A few of the original patients that underwent resurfacing alone without plugging experienced recurrence of symptoms after surgery (37). Since this report, it has been our practice to plug the affected canal in order to obtain a watertight seal with a combination of fascia, bone dust, and bone chips. The middle cranial fossa is then resurfaced with fascia and hydroxyapatite cement. At this time, numerous other groups have reported their series’, with other plugging materials used such as bone wax (11) or bone dust and fibrin glue (90), and with reductions in patient symptoms reported regardless of plugging materials used. Resurfacing material also varies and can include cartilage, fascia, bone dust, fibrin glue, and hydroxyapatite cement. The transmastoid approach to plugging the canal has also been used in many cases with excellent results (38, 90–92). Prior to pursuing surgery, however, control of migraine is critical to avoid exacerbation of migraine after surgery and to distinguish treatable symptoms that are unlikely to be helped by repairing a dehiscence. Jung et al. recently showed patients with migraine have worse dizziness handicap than those without migraine, including after surgery (93).

The selection of surgical approach to repair the dehiscent canal should be based on the patient’s anatomy (42) and on the experience of the treating surgeon. The middle cranial fossa approach is familiar to neurotologists and allows the advantages of directly observing the dehiscent canal and providing assurance that the canal is adequately plugged on either side of the dehiscence. In some cases in which the dehiscence is located adjacent to the superior petrosal sinus or the more posterior aspect of the canal near the common crus, the transmastoid approach is preferred. Alternatively, an angled endoscope may extend visualization for plugging *via* middle cranial fossa approach (94). Furthermore, in some cases the transmastoid approach is not feasible due to a contracted mastoid with a low-hanging tegmen. We feel a significant disadvantage of the transmastoid approach is the lack of directly seeing the dehiscence, thereby risking inadequate plugging on either side of the dehiscence. Some have suggested this can be circumvented by elevating dura over the dehiscence *via* the mastoid and using a mirror to ensure the canal is adequately plugged (92).

Patients generally do well after surgery to plug the affected semicircular canal, with improvements in autophony (9), dizziness handicap (8), and overall health-related quality of life (95). This corresponds with elimination of the third mobile window, as has been demonstrated by the normalization of cervical VEMP thresholds (96), the ocular VEMP amplitudes (96), the elevated SP to AP ratio (86, 89), and the low-frequency air-bone gap (97). After surgical plugging, patients have expected reduction in the function of the superior semicircular canal (7, 98). About one-third of patients have a temporary pan-labyrinthine hypofunction (99), and approximately 15% have plugging material that also impacts function of the posterior semicircular canal (7). Benign paroxysmal positional vertigo has been reported in up to 25% of patients (100). Perhaps surprisingly in the history of otology, exposing and manipulating the membranous labyrinth at surgery only rarely results in a significant loss of hearing, even in patients undergoing revision surgery (97, 101–103). Approximately 25% of patients, however, develop a high-frequency sensorineural hearing loss (97, 104). In our experience, the recurrence rate from plugging and resurfacing of the canal is quite low (77). Furthermore, patients who undergo revision surgery for SCDS tend to do well, but the success rates are lower than in primary surgery.

### Round Window Procedures

Some otologic surgeons have recently begun offering a procedure to reinforce the round window by a variety of methods as an attempt to dampen SCDS symptoms (105, 106). This procedure is proposed as a minimally invasive approach that might provide relief from SCDS symptoms. The proponents argue that stiffening the round window partially dampens one of the three inner ear windows, leaving the oval window and the dehiscence as the primary remaining inner ear windows. Lempert and other proponents of horizontal semicircular canal fenestration invoked a similar philosophy in the early treatment of otosclerosis (107), bypassing the fixed oval window and using instead the round window and a new semicircular canal fenestration to restore compression and rarefaction of inner ear fluids. There have been significant advancements in the understanding of inner ear biomechanics that would suggest that these approaches should induce vertigo, by shunting acoustic energy preferentially across vestibular sensory epithelia. This in fact appears to be the case with complete round window occlusion in two patients with SCDS who subsequently had the process reversed (106).

Furthermore, occlusion of the round window is thought to induce a hearing loss (108, 109). In the series by Silverstein et al., hearing loss was the only subjective survey measure that did not improve after round window reinforcement (106), others have reported a conductive hearing loss (105), and additional series of patients undergoing round window reinforcement by some of the authors have shown a mild hearing loss with this surgery in the non-dehiscent ear (110, 111). Among patients who have had this procedure performed elsewhere, we have observed that if successful, round window reinforcement has provided only temporary relief for SCDS and that some have reported hearing loss and new tinnitus. Whether this transient relief is caused by a reduction in hearing or some other mechanism remains to be determined, and thus far there has not been a proposed model to explain how this alteration in physiology can improve symptoms. We believe this surgery requires additional study before it is recommended to patients.

## Future Directions

There are many interesting remaining questions in SCDS. As described above, the etiology of SCDS remains unknown, in particular an explanation is needed for why patients tend to present later in life if this is primarily a congenital phenomenon. In some cases, symptoms occur after a traumatic incident or sudden increase in intracranial pressure leading to symptom onset. However, many patients do not report an inciting event. It may therefore be some combination of congenital and acquired pathology (112). Identifying the etiology is important for purposes of treatment, for if the etiology can be identified, research toward medical management may become feasible.

Comparative outcomes among surgical approaches are lacking, in part due to a lack of a well-designed disease-specific outcome measure in SCDS to assess for symptom improvement. A validated outcome measure would be a significant step forward in assessing post-intervention outcomes. We believe plugging the canal by middle cranial fossa approach currently is the gold standard for treatment based on the available data. This procedure reduces function of the superior semicircular canal and poses additional risk to the inner ear. While the outcome from canal plugging is supported by reductions in symptoms, improvements in quality of life, and a low risk of recurrence, methods of effectively addressing the pathology without producing impaired inner ear function would be desirable. Perhaps, individually fabricated 3D-printed reconstructions could prevent the third mobile window phenomena without risks of disease recurrence or persistence that have been observed with resurfacing alone (113).

While we know from temporal bone studies and surveys of CT imaging the approximate prevalence of an anatomic dehiscence, we still do not know the prevalence or incidence of SCDS or whether there are modifiable risk factors. For diagnosis, there are many available tests that appear to represent the abnormal pressure transmission associated with a third mobile window (ocular and cervical VEMPs, pure tone audiometry, and ECoG), nevertheless, improved CT imaging techniques such as cone beam or flat panel CT may improve diagnostic accuracy. A recent study observed that some patients with SCDS may also have endolymphatic hydrops as determined by MRI with intratympanic gadolinium (114). This observation needs to be confirmed in a larger, well-defined population, for if confirmed, it may have implications for etiology.

Finally, we have identified some patients with thin, but not frankly dehiscent bone over the superior semicircular canal (i.e., near dehiscence) at the time of surgery (61). These patients often have symptoms of SCDS, physiologic evidence of a third mobile window, and in some cases are noted to have compliance of the thin bone at the time of surgery. On the other hand, these patients tended not to fair as well after surgical repair. It remains to be determined whether patients with thin bone over the superior semicircular canal have poorer outcomes, and additional data are needed.

## Conclusion

Although there are still unanswered questions, superior semicircular canal dehiscence syndrome has become one of the most well-described vestibular disorders. Its physiologic basis is well established through tremendous progress over the last 20 years. This has been the result of a combination of several developments including our collective understanding of novel methods of vestibular testing, development of high-resolution imaging, and pioneering surgeries demonstrating the safety and efficacy of semicircular canal plugging. Consistent diagnostic criteria and a disease-specific outcome measure would allow improved ability to compare treatment outcomes in developing even safer and more enduring therapies.

## Author Contributions

BW reviewed literature and wrote the initial draft. JC and LM performed critical analysis of the initial draft and revisions.

## Conflict of Interest Statement

The authors declare that the research was conducted in the absence of any commercial or financial relationships that could be construed as a potential conflict of interest. The reviewer, ES, and handling Editor declared their shared affiliation, and the handling Editor states that the process nevertheless met the standards of a fair and objective review.
